# Effects of propofol on the development of cancer in humans

**DOI:** 10.1111/cpr.12867

**Published:** 2020-06-29

**Authors:** Yichi Xu, Shuya Pan, Wenxiao Jiang, Fang Xue, Xueqiong Zhu

**Affiliations:** ^1^ Department of Obstetrics and Gynecology The Second Affiliated Hospital of Wenzhou Medical University Wenzhou China

**Keywords:** cancer, miRNAs, prognosis, propofol, signalling pathways

## Abstract

Cancer is one of most the significant threats to human health worldwide, and the primary method of treating solid tumours is surgery. Propofol, one of the most widely used intravenous anaesthetics in surgery, was found to be involved in many cancer‐related pathophysiology processes, mainly including anti‐tumour and minor cancer‐promoting effects in various types of cancer. An increasing number of studies have identified that propofol plays a role in cancer by regulating the expression of multiple signalling pathways, downstream molecules, microRNAs and long non‐coding RNAs. Emerging evidence has indicated that propofol can enhance the anti‐tumour effect of chemotherapeutic drugs or some small molecular compounds. Additionally, in vivo animal models have shown that propofol inhibits tumour growth and metastasis. Furthermore, most clinical trials indicate that propofol is associated with better survival outcomes in cancer patients after surgery. Propofol use is encouraged in cancers that appear to have a better prognosis after its use during surgery. We hope that future large and prospective multicenter studies will provide more precise answers to guide the choice of anaesthetics during cancer surgery.

## Graphical abstract

Propofol inhibits tumor cell proliferation, metastasis, and promotes cell apoptosis and increases the sensitivity to chemotherapeutic drugs. In addition, in vivo animal models have shown that propofol inhibits tumor growth and metastasis. Furthermore, propofol is associated with better survival outcomes in cancer patients after surgery.

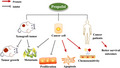

## 1 INTRODUCTION

Cancer‐related death accounts for the majority of deaths among humans in both the developing and developed world. It is estimated that by 2020, in the United States, there will be 1 806 590 new cancer cases and 606 520 cancer deaths.^1^ At present, the primary treatment method for most solid cancers is removing the tumour during surgery. Unfortunately, the surgery process causes a large number of tumour cells to be released, reducing the activity of T, B and NK lymphocytes in the postoperative period, which possibly leads to tumour progression.^2^ Anaesthetic agents may play an essential role in tumour relapse and metastasis because they are administered at the moment of greatest risk of transmission: surgical removal of the tumour.^3^ Recently, many studies have uncovered that the choice of anaesthetic agent could influence the prognosis of cancer patients undergoing cancer surgery.^2^, ^4^, ^5^, ^6^, ^7^, ^8^, ^9^

Propofol (2,6‐diisopropylphenol) is currently one of the most commonly used anaesthetics in clinical practice. It was first introduced into clinical treatment in 1986 and initially served just as an anaesthetic inducer during surgery. However, it soon became one of the most extensively used intravenous anaesthetic agents to produce sedative and anaesthetic effects.^10^ Notably, propofol not only served as a sedative or hypnotic drug during surgery but was also shown to have many non‐anaesthetic effects, such as anti‐tumour or carcinogenic activity, in recent investigations.

In this review, we discuss the effects of propofol on the biological behaviour of cancer and the mechanisms of its biological function in vitro via regulating multiple signalling pathways, downstream molecules, microRNAs (miRNAs) and long non‐coding RNAs (lncRNAs). In addition, we also discuss the pivotal role propofol plays in the inhibition of tumour growth and metastasis in vivo and its inhibitory effect on cancer cells when used in combination with chemotherapeutic drugs or molecular compounds. A series of retrospective and prospective clinical trials have reported the relationship between propofol and patient survival outcomes during tumour surgery.^2^, ^4^, ^5^, ^6^, ^7^, ^8^, ^9^, ^11^, ^12^, ^13^, ^14^, ^15^, ^16^

## 2 ROLE OF PROPOFOL IN CANCER CELLS

### 2.1 Biological behaviour of propofol in cancer cells

Propofol is a popular anaesthetic agent with potent anti‐tumour activity. However, a few studies showed that propofol promoted cell proliferation and invasion in gallbladder cancer and breast cancer.^17^, ^18^ Nevertheless, a large body of literature reports that propofol inhibits the metastasis of cancer cells in colon cancer, breast cancer, lung adenocarcinoma, hepatocellular carcinoma and cervical cancer.^19^, ^20^, ^21^, ^22^, ^23^ Mechanistically, a research group used atomic force microscopy and found that propofol disrupted the cellular cytoskeleton of cervical cancer, which possibly unveiled the biological mechanism of how propofol reduced migration ability.^23^ A study reported that propofol exerted an inhibitory effect on tumorigenesis by promoting autophagic flux and triggering autophagosome accumulation in cervical cancer cells.^24^ Propofol not only inhibited the adhesion and migration of breast cancer cells but also promoted apoptosis.^25^ In oesophageal squamous cell cancer, propofol induced cell apoptosis and reduced proliferation, invasion, and angiogenesis in a dose‐ and time‐dependent manner.^26^ These laboratory data provide support for the tumour‐suppressive effects of propofol in multiple cancers.

Moreover, propofol may play a pivotal role in affecting the tumour microenvironment of the serum in patients undergoing surgery. Recent data showed that in patients undergoing radical resection of non‐small cell lung cancer (NSCLC), the serum of patients treated with propofol had a lower concentration of tumour angiogenesis‐related factors, such as vascular endothelial growth factor (VEGF) and transforming growth factor beta, than that in the sevoflurane group.^27^ Serum of breast cancer patients who were given propofol or sevoflurane during surgery was added to oestrogen receptor (ER)‐negative breast cancer cells, and it was found that more breast cancer cells treated with serum from patients who received propofol underwent apoptosis than those treated with serum from patients who received sevoflurane.^28^ Similarly, serum from colon cancer patients who received propofol during surgery inhibited colon cancer cell proliferation and invasion, and promoted apoptosis in vitro when compared with cells treated using serum from patients who received sevoflurane.^29^ These phenomena indicate that propofol may affect the tumour microenvironment of the serum, thereby playing an inhibitory role in the development and progression of cancer.

### 2.2 Effect of propofol on cancer cells by regulating related signalling pathways or downstream molecules

**FIGURE 1:**
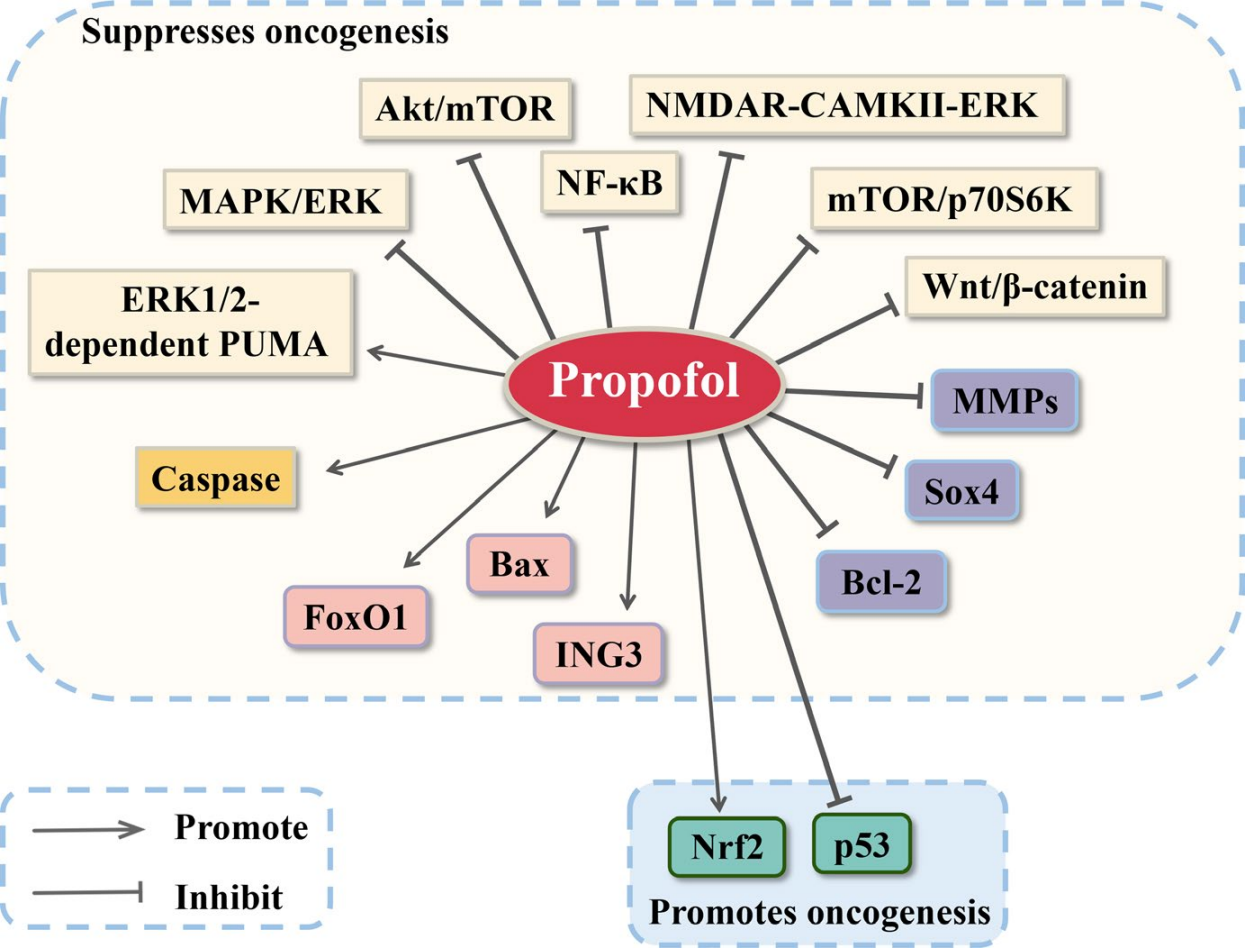
Propofol exerts tumour‐suppressive or oncogenic effects by regulation of related signalling pathways or downstream molecules in cancer cells. Akt, protein kinase B; Bax, B‐cell lymphoma‐2 associated X; Bcl‐2, B‐cell lymphoma‐2; CAMKII, calcium/calmodulin‐dependent protein kinase II; ERK, extracellular signal‐regulated kinases; FoxO1, Forkhead Box O1; ING3, inhibitor of growth 3; MAPK, mitogen‐activated protein kinase; MMP, matrix metalloproteinase; mTOR, mammalian target of rapamycin; NF‐κB, nuclear factor‐kappa B; NMDAR, N‐methyl‐D‐aspartate receptor; Nrf2, nuclear factor E2‐related factor‐2; p53, tumour protein P53; p70S6K, p70 ribosomal protein S6 kinase; Sox4, SRY‐box transcription factor 4; Wnt, wingless and proto‐oncogene integration‐1. “Arrows from propofol to → targets” means “activating targets.” “Blockade from propofol to targets” means “inhibiting targets”

To elucidate the molecular mechanism of the influence of propofol on the biological behaviours of cancer cells, we reviewed a large amount of literature about the regulation of signalling pathways or downstream molecules related to cancer cell proliferation, apoptosis, migration and invasion after propofol stimulation (Table 1; Figure 1). A few studies showed that propofol promoted cell proliferation and invasion in gallbladder cancer in a dose‐dependent and time‐dependent manner by activating the nuclear factor E2‐related factor‐2 (Nrf2) at the transcriptional and translational level.^17^ In accordance with this, propofol induced cell migration by activating the Nrf2 signalling pathway and triggering cell proliferation in part via downregulation of the expression of p53 in human breast cancer MDA‐MB‐231 cells.^18^ Nonetheless, plenty of studies have shown the opposite results. For example, an in vitro study found that propofol suppressed colon cancer cell invasion partly via extracellular signal‐regulated kinases 1 and 2 (ERK1/2)‐dependent downregulation of matrix metalloproteinases (MMPs).^19^ Additionally, to clarify the molecular mechanism in metastatic inhibitory effects of propofol in breast cancer, Li et al^20^ reported that propofol reduced MMP expression via the suppression of nuclear factor‐kappa B (NF‐κB) pathways and inhibited the invasion and migration ability of cancer cells. In cervical cancer, propofol suppressed cell viability and induced apoptosis by inhibiting of the mammalian target of rapamycin (mTOR)/p70 ribosomal protein S6 kinase (p70S6K) pathway.^30^ In glioma cells, propofol inhibited cell growth and increased cell apoptosis by downregulating wingless and proto‐oncogene integration‐1 (Wnt) signalling.^31^ In NSCLC, cell viability was reduced and cell apoptosis was promoted by an increase the activity of the ERK1/2‐dependent PUMA signalling pathway following exposure to propofol.^32^ In cholangiocarcinoma cells, propofol exerted inhibitory effects on metastasis and induced apoptosis. Furthermore, the level of B‐cell lymphoma‐2‐associated X (Bax) expression was increased and B‐cell lymphoma‐2 (Bcl‐2) expression was decreased after adding propofol; the Wnt/β‐catenin signalling pathway was inhibited as the concentration of propofol increased.^33^ In Leydig cell cancer, propofol induced the apoptosis of cells via promoting the activity of caspase, as well as inhibiting the protein kinase B (Akt) pathway.^34^ One study illustrated that propofol inhibited the process of glycolysis and repressed hypoxia‐inducible factor‐1α (HIF‐1α) in colorectal cancer cells via inhibition of the NMDAR‐calcium/calmodulin‐dependent protein kinase II‐ERK pathway, which might be related to the inhibition of cancer progression.^35^ Chen et al^36^ found that propofol might promote cancer cell growth and metastasis via blocking the Wnt/β‐catenin and NF‐κB pathways in an ANRIL‐dependent method in papillary thyroid cancer. Consistent with the previous findings, propofol inhibited the maintenance and self‐renewal of leukaemia stem cells by suppressing Akt/mTOR and Wnt/β‐catenin.^37^ The growth and activity of gastric cancer cells were inhibited by the upregulated expression of the inhibitor of growth 3 (ING3) following treatment with propofol, and ING3 exerted an important role in propofol‐induced anti‐tumour effects.^38^ Notably, propofol inhibited cell proliferation and promoted apoptosis in a dose‐ and time‐dependent manner by upregulating Forkhead Box O1 (FoxO1) expression in oral squamous cell carcinoma. Furthermore, the activity of FoxO1 could be increased at the transcriptional level by binding to the promoter of Growth Arrest Specific 5 after treatment with propofol, thereby enhancing the anti‐tumour effects of propofol.^39^ In addition, under exposure to propofol, the proliferation, migration and invasion of endometrial cancer cells were suppressed due to the downregulated expression of SRY‐box transcription factor 4 (Sox4).^40^ Using cardia cancer cell lines, Su et al^41^ reported that propofol suppressed cell proliferation and induced cell apoptosis, which might be partially associated with the inhibition of the mitogen‐activated protein kinase/ERK signalling pathway. Consistently, propofol elevated the expression of apoptosis‐related proteins such as Caspase‐3, Bax and phosphorylated ERK1/2 in cardia cancer cells. Thus, a number of investigations have uncovered that propofol has inhibitory effects on different cancer cells through various cellular pathways.

**TABLE 1:** The biological function of propofol in cancer cell by regulation signalling pathways or downstream molecules

The role of propofol in oncogenesis	Cancer types	Signalling pathways regulated by propofol	Function	Ref.
Promotes oncogenesis	Gallbladder cancer	Activates Nrf2	Induces cells proliferation and invasion	^17^
	Breast cancer	Activates Nrf2 and downregulates p53	Induces cells migration and proliferation	^18^
Suppresses oncogenesis	Colon cancer	Downregulates MMPs	Inhibits cells invasion	^19^
	Breast cancer	Downregulates MMPs via suppression of NF‐κB pathways	Inhibits cells invasion and migration	^20^
	Cervical cancer	Downregulates mTOR/p70S6K	Inhibits cells viability and induces apoptosis	^30^
	Glioma	Downregulates Wnt pathway	Inhibits cells growth and induces apoptosis	^31^
	Non‐small cell lung cancer	Activates ERK1/2‐dependent PUMA pathway	Inhibits cells viability and induces apoptosis	^32^
	Cholangiocarcinoma	Downregulates Bcl‐2, upregulates Bax and inhibits Wnt/β‐catenin pathway	Inhibits cells metastasis and apoptosis	^33^
	Leydig cell cancer	Activates caspase and inhibits Akt pathway	Induces apoptosis	^34^
	Colorectal cancer	Inhibits NMDAR‐CAMKII‐ERK pathway	Inhibits cells glycolysis and cancer progression	^35^
	Thyroid cancer	Blocks Wnt/β‐catenin and NF‐κB pathways	Promotes cells growth and metastasis	^36^
	Leukaemia stem cell	Suppresses Akt/mTOR and Wnt/β‐catenin	Inhibits cells self‐renewal	^37^
	Gastric cancer	Upregulates ING3	Induces anti‐tumour effects	^38^
	Oral squamous cell carcinoma	Upregulates FoxO1	Inhibits cells proliferation and induces apoptosis	^39^
	Endometrial cancer	Downregulates Sox4	Inhibits cells proliferation, migration and invasion	^40^
	Cardia cancer	Inhibits MAPK/ERK signalling pathway	Inhibits cells proliferation and induces apoptosis	^41^

Abbreviations: Akt, protein kinase B; Bax, B‐cell lymphoma‐2 associated X; Bcl‐2, B‐cell lymphoma‐2; CAMKII, calcium/calmodulin‐dependent protein kinase II; ERK1/2, extracellular signal‐regulated kinases 1 and 2; FoxO1, Forkhead Box O1; ING3, inhibitor of growth 3; MAPK, mitogen‐activated protein kinase; MMP, matrix metalloproteinase; mTOR, mammalian target of rapamycin; NF‐κB, nuclear factor‐kappa B; NMDAR, N‐methyl‐D‐aspartate receptor; Nrf2, nuclear factor E2‐related factor‐2; p53, Tumour Protein P53; p70S6K, p70 ribosomal protein S6 kinase; Sox4, SRY‐box transcription factor 4; Wnt, wingless and proto‐oncogene integration‐1.

### 2.3 Anti‐tumour effect of propofol in cancer cells via regulation of microRNAs

It has been revealed that miRNAs, endogenous non‐coding small RNA molecules, play pivotal roles in affecting gene expression, signalling pathways and cellular biological effects, such as proliferation, metastasis and apoptosis.^42^ Numerous studies have revealed that the effects of propofol in human cancer might be regulated by miRNA expression.

**FIGURE 2:**
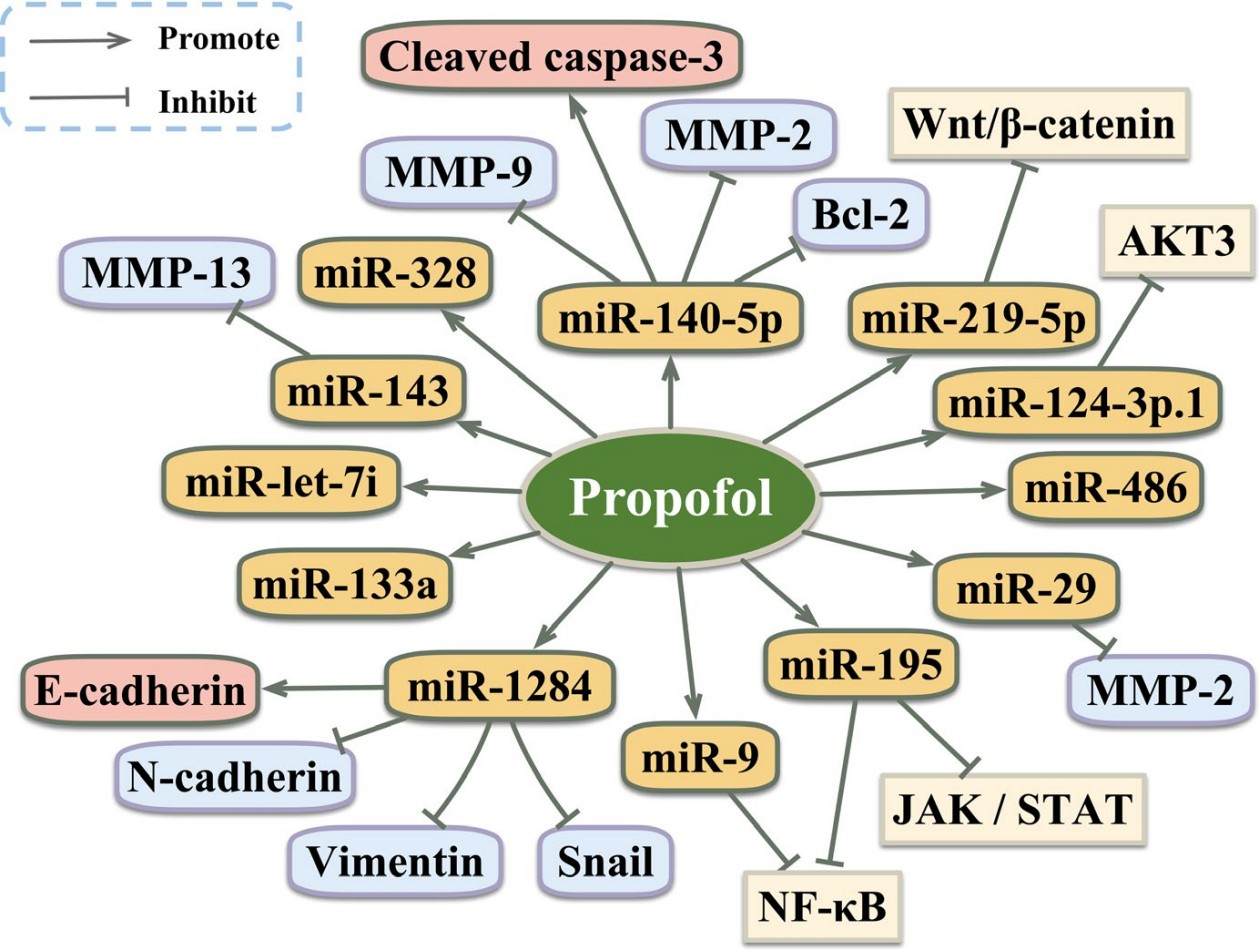
The anti‐tumour effect of propofol by upregulation of miRNAs in cancer cells. AKT, protein kinase B; Bcl‐2, B‐cell lymphoma‐2; JAK, Janus kinase; miR, microRNAs; MMP, matrix metalloproteinase; NF‐κB, nuclear factor‐kappa B; STAT, signal transducer and activator of transcription 3; Wnt, wingless and proto‐oncogene integration‐1. “Arrows from propofol to → targets” means “activating targets.” “Blockade from propofol to targets” means “inhibiting targets”

The expression of miRNAs is elevated after adding propofol, which has tumour‐suppressive action against cancer cells (Table 2; Figure 2). For instance, propofol significantly inhibited epithelial ovarian cancer cell proliferation and promoted apoptosis partly via the upregulation of miR‐let‐7i expression.^43^ In addition, under propofol exposure, the proliferation and invasion of pancreatic cancer cells were effectively inhibited, and apoptosis was triggered in part by the increased expression of miR‐133a.^44^ Using human hepatocellular carcinoma (HCC) cell lines, Gong et al^45^ reported that propofol inhibited the proliferation, invasion and migration of cells by increasing the expression of miR‐219‐5p, and subsequently attenuated the activation of the Wnt/β‐catenin signalling pathway. Propofol significantly suppressed osteosarcoma cell proliferation and invasion, and induced apoptosis, in part due to the decreased expression of MMP‐13 through elevated miR‐143 expression.^46^ In ovarian cancer, propofol inhibited cell invasion and proliferation via upregulating the expression of miR‐9 and suppressing the activity of NF‐κB signalling pathway.^47^ In gastric cancer, propofol has been found to inhibit cell proliferation, migration and invasion by upregulating miR‐195 and subsequent attenuation of the activity of Janus kinase (JAK)/signal transducer and activator of transcription (STAT) and NF‐κB pathways.^48^ Similarly, another study displayed that propofol gradually attenuated gastric cancer cell proliferation, migration and invasion in a time‐ and dose‐dependent manner, which was in line with findings from the previous studies mentioned above.^49^ It is worth noting that propofol exerted these anti‐tumour effects in gastric cancer cells by upregulating miR‐29 family members and downregulating its target gene, MMP‐2.^49^ Additionally, propofol elevated the expression of cleaved caspase‐3 and decreased the expression of Bcl‐2, MMP‐2 and MMP‐9 by upregulating miR‐140‐5p expression in gastric cancer cells, resulting in the decrease of cancer cell proliferation, migration, and invasion and the increase of cell apoptosis.^50^ In NSCLC, propofol significantly inhibited cell proliferation and promoted cell apoptosis through upregulation of miR‐486.^51^ Furthermore, the growth, migration, invasion and epithelial‐mesenchymal transition (EMT) process of NSCLC cells were inhibited following exposure to propofol through the upregulated expression of miR‐1284, thereby increasing the expression of E‐cadherin and downregulating N‐cadherin, vimentin and Snail.^52^ Additionally, it was found that propofol exerted striking inhibitory effects on cell growth and metastasis by enhancing the expression of miR‐328 in pancreatic cancer.^53^ Moreover, Li et al^54^ found that propofol inhibited the proliferation, migration and invasion of colorectal cancer cells by increasing the expression of miR‐124‐3p.1 and decreasing that of its target gene, AKT3. Another essential result of the study was that the inhibitory anti‐tumour effects of propofol on colorectal cancer cells could be reversed by suppressing miR‐124‐3p.1 expression or the overexpression of AKT3.

**TABLE 2:** The biological function of propofol in cancer cell by regulation of miRNA or lncRNA

miRNA/lncRNA	Cancer types	miRNA or lncRNA regulated by propofol	Downstream gene regulated by miRNA or lncRNA	Functions	Ref.
miRNA	Epithelial ovarian cancer	Upregulates miR‐let‐7i	/	Inhibits cells proliferation and induces apoptosis	^43^
	Pancreatic cancer	Upregulates miR‐133a	/	Inhibits cells proliferation and invasion	^44^
	Hepatocellular carcinoma	Upregulates miR‐219‐5p	Inactivates Wnt/β‐catenin signalling pathway	Inhibits cells proliferation and metastasis	^45^
	Osteosarcoma	Upregulates miR‐143	Downregulates MMP‐13	Inhibits cells proliferation and invasion; induces cells apoptosis	^46^
	Ovarian cancer	Upregulates miR‐9	Downregulates NF‐κB signalling pathway	Inhibits cells invasion and proliferation	^47^
	Gastric cancer	Upregulates miR‐195, miR‐29 family and miR‐140‐5p	Downregulates JAK/STAT, NF‐κB pathways, MMP‐2, MMP‐9 and Bcl‐2; Upregulates cleaved caspase‐3	Inhibits the cell proliferation, migration and invasion; induces cell apoptosis	48, 49, 50
	Non‐small cell lung cancer	Upregulates miR‐486 and miR‐1284	Upregulates E‐cadherin; downregulates N‐cadherin, vimentin and Snail	Inhibits the cell proliferation, metastasis and epithelial‐mesenchymal transition process; promotes apoptosis	51, 55
	Pancreatic cancer	Upregulates miR‐328	/	Inhibits cells growth and metastasis	^53^
	Colorectal cancer	Upregulates miR‐124‐3p.1	Downregulates AKT3	Inhibits cells proliferation and metastasis	^54^
	Hepatocellular carcinoma	Downregulates miR‐374a	Inhibits Wnt/β‐catenin and PI3K/ AKT pathways	Inhibits cell viability, migration and invasion; promotes apoptosis	^55^
	Gastric cancer	Downregulates miR‐221	/	Inhibits the proliferation and invasion; promotes apoptosis	^56^
	Pancreatic cancer	Downregulates miR‐21	Inhibits Slug pathway; increases PUMA and E‐cadherin	Inhibits cell growth and invasion	^57^
	Breast cancer	Downregulates miR‐21 and miR‐24	Inactivates PI3K/AKT, Wnt/β‐catenin and p27	Inhibits cells proliferation and EMT; promotes apoptosis	58, 59
	Non‐small cell lung cancer	Downregulates miR‐372	Inactivates Wnt/β‐catenin and mTOR signalling pathways	Inhibits cells growth, migration and invasion	^60^
	Hepatocellular carcinoma	Downregulates miR‐142‐3p	Downregulates RAC1	Inhibits cells metastasis	^62^
lncRNA	Colon cancer	Downregulates HOTAIR	Inactivates Wnt signalling pathway	Inhibits cell metastasis and promotes cell apoptosis	^65^
	Colorectal cancer	Downregulates HOXA11‐AS	Upregulates miR‐let‐7i	Promotes cell apoptosis	^66^
	Gastric cancer	Downregulates MALAT1	Downregulates ATG5	Promotes cell apoptosis and reduces chemoresistance	^67^

Abbreviations: AKT, protein kinase B; ATG5, autophagy‐related genes 5; Bcl‐2, B‐cell lymphoma‐2; HOTAIR, HOX antisense intergenic RNA; HOXA11‐AS, HOXA11 Antisense RNA; JAK, Janus kinase; MALAT1, metastasis‐associated lung adenocarcinoma transcript 1; MMP, matrix metalloproteinase; mTOR, mammalian target of rapamycin; NF‐κB, nuclear factor‐kappa B; p27, cyclin‐dependent kinase inhibitor 1B; PI3K, phosphoinositide 3‐kinase; RAC1, Ras‐related C3 botulinum toxin substrate 1; STAT, signal transducer and activator of transcription 3; Wnt, wingless and proto‐oncogene integration‐1.

**FIGURE 3:**
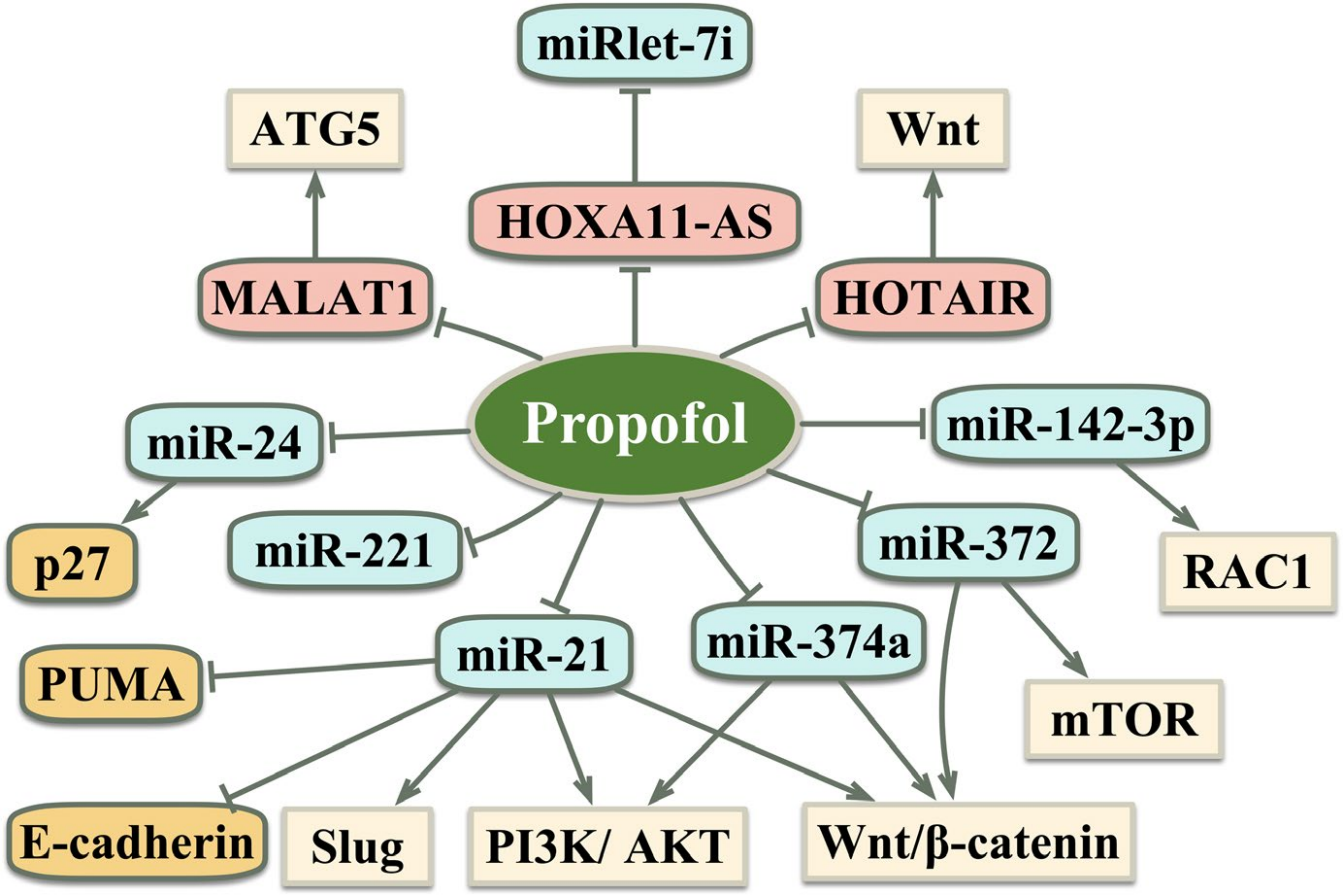
The anti‐tumour effect of propofol by downregulation of miRNAs or lncRNAs in cancer cells. AKT, protein kinase B; ATG5, autophagy‐related genes 5; HOTAIR, HOX antisense intergenic RNA; HOXA11‐AS, HOXA11 Antisense RNA; MALAT1, metastasis‐associated lung adenocarcinoma transcript 1; miR, microRNAs; mTOR, mammalian target of rapamycin; p27, cyclin‐dependent kinase inhibitor 1B; PI3K, phosphoinositide 3‐kinase; RAC1, Ras‐related C3 botulinum toxin substrate 1; Wnt, wingless and proto‐oncogene integration‐1. “Arrows from propofol to → targets” means “activating targets.” “Blockade from propofol to targets” means “inhibiting targets”

A large number of miRNAs act as a driving force in the progression of cancer and their expression levels decrease after adding propofol to cancer cells, which may explain the anti‐tumour effects of propofol (Table 2; Figure 3). For example, propofol contributed to inhibiting cell viability, migration, and invasion, and promoting apoptosis by downregulating miR‐374a and then inhibiting the Wnt/β‐catenin and phosphoinositide 3‐kinase (PI3K)/AKT pathways in HCC.^55^ Similarly, propofol inhibited the proliferation and invasion of gastric cancer cells, and promoted apoptosis partly via decreased expression of miR‐221.^56^ Propofol inhibited cell growth and invasion by suppressing the miR‐21/Slug pathway in pancreatic cancer, leading to increased expression of PUMA and E‐cadherin.^57^ A recent study found that under propofol treatment, the number of apoptotic cells was increased via inhibition of the miR‐24/cyclin‐dependent kinase inhibitor 1B (p27) signalling pathway in breast cancer.^58^ Moreover, it has been pointed out that propofol attenuated cell proliferation and EMT by downregulating miR‐21 expression and then inactivating the PI3K/AKT and Wnt/β‐catenin pathways in breast cancer.^59^ Sun et al^60^ reported that propofol inhibited NSCLC cell growth, migration and invasion by downregulating miR‐372 expression and subsequently suppressed the activity of the Wnt/β‐catenin and mTOR pathways.

A recent study discovered that microvesicles were able to shuttle miRNAs into nearby or distant cells and then regulate the expression of target genes.^61^ Zhang et al^62^ showed that propofol triggered the shutting of miR‐142‐3p from macrophages to HCC cells, which decreased the expression of Ras‐related C3 botulinum toxin substrate 1 (RAC1) and then inhibited the metastasis of HCC cells. Furthermore, extracellular vesicles and their microRNA cargo are important promoters of malignant cell communication and mediate the effects of anaesthetics on tumour biology during colorectal cancer surgery. Buschmann et al^63^ found that propofol influenced microRNA profiles in extracellular vesicles in serum samples and affected the signal pathways involved in the inhibitory effect of proliferation and metastasis in colorectal cancer patients receiving surgery.

Taken together, these investigations revealed that propofol contributes to triggering anti‐tumour activity by downregulating or upregulating miRNA expression and can be used as a treatment agent in various cancers.

### 2.4 Effect of propofol on cancer cells due to the regulation of long non‐coding RNAs

LncRNAs are longer than 200 nucleotides but are unable to express proteins. It was emphasized that lncRNAs play an essential role in the occurrence and progression of cancer.^64^ One study revealed that propofol decreased colon cancer cell metastasis and increased cell apoptosis in part through the inhibition of lncRNA HOX antisense intergenic RNA expression (HOTAIR) and subsequent inactivation of the Wnt signalling pathway.^65^ The potential mechanism of propofol in colorectal cancer is the suppression of tumorigenesis by downregulating a highly conserved lncRNA HOXA11 Antisense RNA (HOXA11‐AS) and upregulating miR‐let‐7i, and then promoted cell apoptosis.^66^ Additionally, propofol also plays an essential role in regulating autophagy.^67^ To date, autophagy has been found to play a dual role in the development of cancer dependent on a variety of factors including the tumour microenvironment, cancer type and stage and genetic background.^68^ In the early stages of tumorigenesis, autophagy acted as a tumour suppressor, which prevented tumour initiation, proliferation, invasion and metastasis.^69^, ^70^, ^71^ However, once the tumours progress to the late stage, autophagy promoted tumorigenesis by sustaining tumour metabolism, growth, survival, and facilitating metastasis and resistance to therapeutic agents.^72^, ^73^, ^74^ In gastric cancer cells, propofol facilitated apoptosis and reduced autophagy‐related chemoresistance to cisplatin via inhibition of lncRNA metastasis‐associated lung adenocarcinoma transcript 1 (MALAT1). Mechanically, it has been demonstrated that the level of MALAT1 was lower and miR‐30e was upregulated, which caused suppression of autophagy‐related genes 5 (ATG5), and subsequently inactivated autophagy‐related chemoresistance in gastric cancer cells following propofol treatment.^67^ These investigations have shown that propofol exerted tumour‐inhibitory effects by regulating lncRNA expression (Table 2; Figure 3).

### 2.5 Effect of propofol in combination with chemotherapeutic drugs or molecular compounds in cancer cells

Recently, more studies have found that the combination of propofol and chemotherapeutic agents or some molecular compounds may play anti‐tumour roles in human cancer (Table 3). For instance, propofol enhanced the chemosensitivity to cisplatin‐induced apoptosis in cervical cancer cells through the epidermal growth factor receptor (EGFR)/JAK2/STAT3 pathway.^75^ In ovarian cancer cells, treatment with propofol and cisplatin promoted cell apoptosis by elevating the activity of the FOXO1 pathway.^76^ Consistent with this study, all paclitaxel‐sensitive and paclitaxel‐resistant cells underwent paclitaxel‐induced apoptosis via decreased expression of the Slug protein under treatment with propofol in ovarian cancer.^77^ A further study found that propofol reduced the upregulation of the NF‐κB signalling pathway induced by gemcitabine, thereby promoting the chemosensitivity of pancreatic cancer cells to gemcitabine.^78^ Additionally, it was widely accepted that hypoxia is a common phenomenon in tumour development and related to the EMT process and cancer progression.^79^ Propofol reversed the drug resistance of docetaxel induced by hypoxia and attenuated the process of EMT via inhibition of HIF‐1α in prostate cancer cells, suggesting that propofol may help overcome prostate cancer's resistance to chemotherapy under hypoxic conditions.^80^ In addition, propofol effectively inhibited lipopolysaccharide (LPS)‐induced EMT and migration in NSCLC cells. Consistently, propofol suppressed the invasion of NSCLC cells by downregulating the expression of HIF‐1α in LPS‐stimulated NSCLC cells.^81^

**TABLE 3:** The anti‐tumour effects of propofol in combination with chemotherapy drugs or molecular compounds in cancer cells

Chemotherapy drugs or molecular compounds	Cancer types	Downstream gene regulated by propofol	Functions	Ref.
Cisplatin	Cervical cancer	EGFR/JAK2/STAT3 pathway	Enhances the chemosensitivity	^75^
Cisplatin	Ovarian cancer	Activates FOXO1	Promotes cell apoptosis	^76^
Paclitaxel	Ovarian cancer	Downregulates Slug	Promotes cell apoptosis	^77^
Gemcitabine	Pancreatic cancer	Downregulates NF‐κB signalling pathway	Enhances the chemosensitivity	^78^
Docetaxel	Prostate cancer	Downregulates HIF‐1α	Enhances the chemosensitivity	^80^
LPS	Non‐small cell lung cancer	Downregulates HIF‐1α	Inhibits EMT, migration and invasion	^81^

Abbreviations: EGFR, epidermal growth factor receptor; EMT, epithelial‐mesenchymal transition; FOXO1, Forkhead Box O1; HIF‐1α, hypoxia‐inducible factor‐1α; JAK2, Janus kinase 2; LPS, lipopolysaccharide; NF‐κB, nuclear factor‐kappa B; STAT3, signal transducer and activator of transcription 3.

Herein, the significantly suppressive role of propofol in combination with chemotherapeutic drugs or molecular compounds was observed in propofol‐treated cancer cells, supporting the anti‐tumour activity of propofol.

## 3 EFFECT OF PROPOFOL ON ANIMAL MODELS OF CANCER

Various investigations have discovered that propofol has essential anticancer properties not only in vitro but also in vivo. A large number of studies indicated that in nude mice models, propofol inhibited the growth of xenograft tumours in numerous cancers such as cervical cancer, lung cancer and pancreatic cancer.^30^, ^82^, ^83^ The latter results appear to be consistent with previous studies that found propofol decreased the growth of endometrial cancer in a xenograft tumour model, but the effect was abolished by increased expression of Sox4.^40^ Xu et al^31^ demonstrated that propofol decreased the growth of tumour and inhibited the Wnt signalling pathway by inhibiting the expression of Wnt target genes (such as *AXIN2*, *CCND1* and *BCL2*) in glioma. In addition, the growth and volume of tumour in mice decreased remarkably under treatment with propofol in colorectal cancer, but the inhibitory effect could be reversed by rapastinel, an agonist of the N‐methyl‐D‐aspartate (NMDA) receptor.^35^ Combining cisplatin and propofol, Zhang et al^67^ demonstrated that the size and weight of the gastric cancer xenograft model were significantly decreased compared with that of the control group, which indicated that propofol increased the chemosensitivity of gastric cancer to cisplatin. Furthermore, under propofol and cisplatin treatment, the inhibitory effect on tumour growth of gastric cancer xenograft was strengthened by downregulation of MALAT1 and ATG5 expression, which might be partly achieved via inhibition of autophagy. In a recent study, propofol anaesthesia or sevoflurane anaesthesia was used for breast cancer resection in an orthotopic mouse model. It was found that the propofol treatment group had lesser lung metastasis than the sevoflurane group 2 weeks after surgery.^84^ Another important result of the study was that the IL‐6 and VEGF serum levels were significantly lower in the propofol group than in the sevoflurane group.^84^ Consistent with in vitro studies, results of these explorations in vivo also suggest that propofol exerts an inhibitory effect on tumour growth and metastasis, as well as facilitates the sensitivity of tumours to cisplatin.

## 4 EFFECT OF PROPOFOL ON THE PROGNOSIS OF PATIENTS WITH CANCER

A number of clinical investigations found that propofol has no effect on prognosis in several cancers (Table 4). A number of retrospective studies have indicated that propofol did not influence survival outcomes in other types of cancer following surgery. For example, in a retrospective cohort study, there was no difference in mortality or locoregional recurrence rate in a 5‐year follow‐up after treatment with desflurane or propofol during breast cancer surgery.^11^ Consistent with previous investigations, Cata et al^12^ reached the conclusion that propofol was not a protective factor for cancer recurrence during breast cancer surgery relative to sevoflurane anaesthesia. In NSCLC, a retrospective study revealed that there was no difference between the propofol group and the inhalation group for overall survival (OS) or relapse‐free survival (RFS) during lung cancer surgery.^13^ Recently, one study found that in patients with high‐grade glioma undergoing cancer surgery, intraoperative use of propofol or sevoflurane anaesthesia led to no difference in OS or progression‐free survival (PFS).^14^ In the study by Oh et al,^15^ among patients who underwent gastric cancer surgery, there was no difference in cancer‐related mortality after using propofol or inhalation anaesthesia. Nonetheless, these findings are retrospective and have some important limitations. Recently, consistent with previous studies, a randomized controlled trial including more than 2000 women undergoing breast cancer surgery reported that breast cancer recurrence did not reduce in paravertebral blocks or in the propofol group compared with the volatile anaesthetic and opioids for analgesia groups.^16^ These results have demonstrated that the prognosis of these cancer patients undergoing cancer surgery is no different than the prognosis of those using other volatile anaesthetics.

**TABLE 4:** The effects of propofol on the prognosis of cancer patients compared with volatile anaesthesia

Type of study	Cancer types	Volatile anaesthesia	Outcomes	Ref.
Retrospective	Breast cancer	Desflurane or sevoflurane	No difference	11, 12
Retrospective	Non‐small cell lung cancer	Sevoflurane	No difference	^13^
Retrospective	High‐grade glioma	Sevoflurane	No difference	^14^
Retrospective	Gastric cancer	Desflurane or sevoflurane	No difference	^15^
Prospective	Breast cancer	Sevoflurane	No difference	^16^
Retrospective	Oesophageal cancer	Isoflurane, sevoflurane or desflurane	Better OS and RFS	^4^
Retrospective	Gastric cancer	Sevoflurane	Better OS	^5^
Retrospective	Colon cancer	Desflurane	Better OS and DFS; less postoperative metastasis	^6^
Retrospective	Hepatocellular carcinoma	Desflurane	Better OS	^7^
Retrospective	Cholangiocarcinoma	Desflurane	Better survival and less postoperative metastases	^8^
Prospective	Bladder cancer	Sevoflurane	Longer DFS	^2^

Abbreviations: DFS, disease‐free survival; OS, overall survival; RFS, recurrence‐free survival.

However, many studies, including retrospective investigations and prospective trials, have evaluated the relationship between propofol and prognosis thus far and concluded that propofol treatment can improve survival in many cancer patients (Table 4). In oesophageal cancer, a retrospective investigation showed that treatment with propofol during surgery was related to better 1‐, 3‐ and 5‐year OS rates and RFS in comparison with the use of volatile anaesthetic agents (including isoflurane, sevoflurane and desflurane).^4^ Similarly, a retrospective study included a total of 2856 patients who received gastric cancer resection surgery and found that the OS of the patients in the propofol group was better than that of the sevoflurane group.^5^ Colon cancer patients treated with propofol exhibited a favourable OS and disease‐free survival (DFS) and less postoperative metastasis than those who were treated with desflurane during surgery.^6^ Compared with desflurane, propofol was associated with better OS when used in hepatectomy for HCC. Moreover, patients with HCC had lower cancer‐specific mortality, and less distant metastasis and local recurrence in the propofol group than in the desflurane group.^7^ Consistent with those results, propofol anaesthesia was related to better survival and less postoperative metastases in comparison with desflurane anaesthesia in open intrahepatic cholangiocarcinoma surgery.^8^ Notably, a meta‐analysis study including 7866 patients with different types of cancer discovered that the use of propofol improved the OS and RFS of cancer in comparison with the use of inhalational anaesthesia.^9^ Additionally, another retrospective cohort study found that propofol anaesthesia was related to better OS and a lower postoperative biochemical recurrence rate than those with desflurane anaesthesia in patients undergoing robot‐assisted radical prostatectomy.^85^ However, these previous studies have the limitations of most retrospective investigations. Hence, a prospective trial was warranted to evaluate the effect of propofol on bladder cancer outcomes. There were 100 patients who underwent radical cystectomy, and it was found that patients treated with the combination of propofol and epidural anaesthesia had a longer DFS than patients who were treated with sevoflurane combined with opioids.^2^ There is growing evidence suggesting that volatile anaesthesia may contribute to poor survival outcomes, whereas propofol seems to provide better survival outcomes in cancer patients undergoing tumour removal surgery.

A series of studies have shown that the different influences of propofol on oncogenic outcomes might depend on the primary site of cancer. However, most studies still show that propofol contributes to a better prognosis. It is encouraged to use propofol in cancers that appear to have a better prognosis after using propofol during the surgery. For cancers in which the prognosis was not influenced by propofol, prospective multicenter studies with larger sample sizes are currently underway, which are expected to provide clear answers.

## 5 CONCLUSION

In summary, the role of propofol in the progression of cancer patients is relatively consistent across a large number of laboratory researches and clinical investigations. Only a few results suggest that propofol increases cell proliferation in certain cancer types and several clinical trials indicated that propofol has no influence on the prognosis of cancer patients. However, most in vitro studies suggest that propofol inhibits most tumour cell proliferation, migration and invasion, and promotes cell apoptosis by regulating many signalling pathways and non‐coding mRNA expression. Consistent with this, numerous in vivo studies indicated that propofol suppresses tumour growth and metastasis. Additionally, a large body of literature has indicated that propofol is associated with better survival outcomes in cancer patients after surgery relative to the use of volatile anaesthetics. Propofol also exerts significant effects in increasing the sensitivity to chemotherapeutic drugs.

Therefore, it is critical to elucidate how to choose the most suitable anaesthetic agents for different types of cancer to obtain the best prognosis for cancer patients. Propofol use is encouraged in cancers that appear to have a better prognosis after its use during the surgery. For cancers in which the prognosis was not influenced by propofol, further animal trials and prospective clinical studies are needed to understand the effects that the type of anaesthesia has on the development of cancer and provide us with more precise answers to guide the choice of anaesthetics during cancer surgery.

## CONFLICT OF INTEREST

The authors declare that they have no conflict of interest.

## AUTHOR CONTRIBUTIONS

YX, WJ and XZ conceptualized and design the manuscript. YX and SP searched the literature and wrote the manuscript. FX, WJ and XZ critically viewed, edited and approved the manuscript. All authors read and approved the final manuscript.

## Data Availability

The data that support the findings of this study are available from the corresponding author upon reasonable request.
